# Scientific Items

**Published:** 1885-02-20

**Authors:** 


### SCIENTIFIC ITEMS.

**Winds and the Weather.**—The Farmers' Club of the American Institute has issued the following rules for foretelling the weather. If farmers, and others whose business is out-doors and depends upon the weather, will study them closely they will be able to guess the weather more accurately than Wiggins or Vennor:

1. When the temperature falls suddenly there is a storm forming south of you.

2. When the temperature rises suddenly there is a storm north of you.

3. The wind always blows from a region of fair weather toward a region where a storm is forming.

4. Cirrus clouds always move from a region where a storm is in progress to a region of fair weather.

5. Cumulous clouds always move from a region of fair weather to a region where a storm is forming.

6. Where cirrus clouds are moving rapidly, from the north or northeast, there will be rain inside of twenty-four hours, no matter how cold it is.

7. When cirrus clouds are moving rapidly, from the south or southeast, there will be a cold rain storm on the morrow ; if it be in the winter, there will be a snow-storm.

8. The wind always blows in a circle around a storm, and when it blows from the north, the heaviest rain is east of you ; if it blows from the south, the heaviest rain is west of you ; if it blows from the east, the heaviest rain is south of you ; if it blows from the west, the heaviest rain is north of you.

9. The wind never blows unless rain or snow is falling within 1,000 miles of you.

10. Whenever heavy white frost occurs, a storm is forming within 1,000 miles north or northwest of you.

As some of our readers may not know what is meant by the two kinds of clouds mentioned above, we give a brief explanation. Cirrus clouds are those which have a thin, streaming look, sometimes appearing like hair or carded wool, and sometimes like a brush or broom. They are the highest of all the clouds. Cumulus clouds are those which appear in round rolling masses; piled one above another, and sometimes looking like great mountains topped with snow.

**The Oil-Spot.**—About ten miles to the south of the Sabine River, which forms the boundary between Texas and Louisiana, and about a mile from the shore, there exists a natural phenomenon known to sailors as "The Oil-Spot." In fine weather there is nothing remarkable to attract the attention of a stranger ; but when an angry gale from the northeast sweeps the ocean, and great crested waves rise in battle array, this charmed natural harbor reveals itself. No visible boundary divides it from the tempestuous ocean around; but, within a space of two miles in length, the waters remain perfectly calm, their only change being that they become turbid and red, as though the oil-bearing mud were stirred up from below. A broad belt of white foam and towering breakers marks where the mighty waves, rolling shoreward in their might, with all the force gathered in an unbroken sweep of 700 miles across the Gulf, are suddenly arrested, and sink down, conquered and powerless, so soon as they come within the mysterious influence of this gentlest of rulers.

Unfortunately, this peaceful haven is very shallow ; its depth is variously stated at twelve and eighteen feet, so that only vessels of light burden can here take shelter. But to these, blessed, indeed, is the change of passing suddenly from the wild tossing of the outer ocean to the wonderful calm of this strange harbor, where the weary crew may rest as securely as though within an encompassing coral reef. Indeed, the stranger approaching this wall of breakers would naturally assume it to be caused by a dangerous reef, and would, as a matter of course, seek safety by steering away from it.

We believe that no scientific examination of this so-called Oil-Spot has yet been made. Sailors who have here found refuge state that the bottom is a soft, soapy mud, into which they can easily push a pole to a considerable depth—a mud, which, when applied to deck-scrubbing, is found to be exceedingly cleansing.—*Popular Science Monthly.*

**A New Application of the Electric Light.**—At the recent electrical exhibition in Philadelphia, Dr. R. W. St. Clair, of Brooklyn, N. Y., exhibited an electrical lamp, which is intended for the use of dentists and surgeons in lighting up the mouth, or other cavities of the body. The lamp is made on the same principle as that of the ordinary incandescent light ; but the glass globe is made extremely small, in some cases not larger than a pea ; and the light is produced by the incandescence of a filament of carbon, formed, it is said, from the vein of a beech-leaf. With such a small lamp, a powerful current is not necessary ; and the doctor has devised a battery which will keep the lamp lighted for several hours before it is necessary to renew the chemicals. The uses of such a lamp are very numerous, and will at once suggest themselves to a physician ; and it will doubtless be of great value to dentists in lighting up the cavity of the mouth during difficult dental operations.—*Popular Science News.*

**Snow-Water Impurities.**—Under the heading of "The Beautiful Snow," the Microscope points out the kind of organic impurities found in snow, which very conclusively shows the fallacy of the idea that melted snow forms a good substitute for distilled water. The impurities are as follows : Living infusoria and algæ, baccilli, and micrococci, mites, diatoms, and great numbers of fungi spores ; also, fibres of wood, mouse hairs, pieces of butterfly wings, skin of larvæ of insects, cotton fibres, pieces of grass, epidermis, pollen grains, rye and potato flour, grains of quartz, minute pieces of roofing tile, and bits of iron and coal.—*Scientific American.*

M. Le Baron Larrey recently deposited with the Paris Academy of Medicine a sealed letter containing details of a new stethescope, invented by M. Gavoy. The instrument is understood to be a little telephone, which amplifies, without exaggeration, the thoracic sounds, both pulmonary and cardiac.—*Ib.*

